# The Relationship between Cognitive Function, Lifestyle Behaviours and Perception of Stress during the COVID-19 Induced Confinement: Insights from Correlational and Mediation Analyses

**DOI:** 10.3390/ijerph18063194

**Published:** 2021-03-19

**Authors:** Hela Znazen, Maamer Slimani, Nicola Luigi Bragazzi, David Tod

**Affiliations:** 1 Department of Physical Education and Sport, College of Education, Taif University, P.O. Box 11099, Taif 21944, Saudi Arabia; znazen.hela@laposte.net; 2 Department of Health Sciences (DISSAL), Postgraduate School of Public Health, Genoa University, 16132 Genoa, Italy; 3 Laboratory for Industrial and Applied Mathematics (LIAM), Department of Mathematics and Statistics, York University, Toronto, ON M3J 1P3, Canada; robertobragazzi@gmail.com; 4 School of Sport and Exercise Science, Liverpool John Moores University, Liverpool L3 3AF, UK; D.A.Tod@ljmu.ac.uk

**Keywords:** COVID-19, stress, lifestyle, cognition, confinement

## Abstract

**Background**: Home confinement during the COVID-19 outbreak may affect lifestyle behaviours, such as daily physical activity, social relationships, eating behaviours, and sleep, among others, which in turn may compromise mental health and psychological states. The aim of the present study was to determine the effects of COVID-19-induced home confinement on stress, attention, and lifestyle behaviours and the correlations between them. **Methods**: Participants included 144 students (aged 18–22 years, 62.5% female, 89.5% single). Stress, attention, and lifestyle behaviours were assessed using the Perceived Stress Scale (PSS), d2 test, and the Simple Lifestyle Indicator Questionnaire adapted and modified (SLIQ), respectively. Total PSS score, concentration performance (CP), errors (E), and lifestyle behaviours (e.g., diet, exercise/activity, alcohol, and smoking) before and during confinement were calculated. **Results**: The data showed a significant difference between before and during confinement in total PSS, CP, E, and all lifestyle behaviours (all, *p* < 0.05). Significant correlations existed between total PSS score, CP, E, and lifestyle behaviours (r= −87–98, all, *p* < 0.05). **Conclusions**: Home confinement has a negative effect on stress, attention, and lifestyle behaviours. This study suggests that the adoption of proper lifestyle behaviours, particularly diet, disciplined hygiene, and physical activity, boost health, psychological states, and cognitive function during COVID-19-induced confinement.

## 1. Introduction

The “Coronavirus disease 2019” (COVID-19) pandemic caused by the “Severe Acute Respiratory Syndrome-related Coronavirus type 2” (SARS-CoV-2) represents the global crisis of our times, which first appeared in the city of Wuhan on 17 November 2019, in Hubei province (central China), before spreading out around the world. The diffusion of this pandemic has triggered a series of exceptional measures to curb the transmission of the virus: the shutting down of public places, businesses and schools, closures of borders between countries, restrictions on mobility and access to certain regions/territories, massive use of teleworking, strict lockdown, and the banning of all organized and social gatherings. These measures have allowed us to “flatten the curve” of the progression of the disease, and to curtail the toll of daily deaths in some countries, although other countries and states are experiencing a sharp increase in cases where the measures have been lifted/eased too early or even abandoned. On the other hand, these measures have had a high cost on economic activity around the world.

Despite the positive effect of these measures on the COVID-19 outbreak, they have had an adverse effect on lifestyle behaviours, such as diet, social relationship, physical activity, and sleep [1], causing bad hygiene and increased sedentary time. In addition, some measures may also increase the time people spend thinking about the pandemic and on social media, which in turn may increase the risk for developing some disorders related to poor diets and lack of sleep, such as anorexia, bulimia, cardiovascular disease, and obesity [2]. All these consequences have a dramatic impact on mental health and psychological states.

The pandemic and the associated confinement have caused a situation that can be described as extreme and, to some extent, even unprecedented. Pérez-Rodrigo et al. [3] reported that dietary habits significantly changed during the confinement in Spanish adults in a negative direction. Confinement can be accompanied by symptoms of anxiety, depression and negative emotions [4], which are risk factors for food restriction, emotional feeding, and hyperphagic access [5,6,7]. To date, all aspects of people’s lives have been profoundly affected and impaired, resulting in a marked uncertainty and making people become aware of the fragility of life. Amongst these aspects of people’s lives, of paramount importance are their psychological states [1]. On the other hand, Di Renzo [8] found improved dietary habits during the COVID-19-induced restrictions: a sample of 3533 Italian participants aged 12–86 years, 76.1% of which were females, tended to adhere to the Mediterranean diet, especially those between 18 and 30 years of age, with an increased uptake of organic foods, including fruits and vegetables. According to a study conducted in Kuwait, in a sample of 415 participants aged 18–73 years, changes in eating practices could be reported, including reduced consumption of fast-food and junk food, and increased ingestion of fresh, healthy food, such as fish and seafood [9]. During confinement, people have greater access to food because they work and live in closer proximity to their homes and kitchens. Greater access during a stressful situation likely leads to increased emotional, uncontrolled eating, and depressed mood [10]. Additionally, increased exposure to food advertisements (through increased media exposure) may be accompanied by people experiencing increased cravings for food and weight gain, both short and long term [11]. Behavioural changes may be complex, reflecting attempts and efforts to cope with rather challenging and unprecedented situations [12].

The clinical impact is likely to be greater when individuals or their families have health problems that are also some of the risk factors for developing SARS-CoV-2 infection (e.g., being overweight or obese, smoking history, suffering from chronic-degenerative disorders or other underlying co-morbidities, such as hypertension, cardiovascular disease, and diabetes, among others) [13,14,15]. Furthermore, time spent thinking about the pandemic, media exposure, the consumption of misleading news, and misinformation are also aggravating factors that exacerbate psychological distress. Therefore, it can be anticipated that the burden imposed by anxiety, depression, and sleep disorders will be on the rise. Moreover, some scholars have described new COVID-19 specifically related symptoms, which appear to be associated with virus contamination, such as COVID-19-induced post-traumatic stress disorder (PTSD) or “post-COVID stress disorder” [4,16].

However, although the impact of COVID-19 on health has been explored during the first year of the pandemic, a thorough examination of the effects of home confinement remains scarce, especially in terms of the relationship between cognitive, lifestyle, and stress variables during confinement. Therefore, the aim of the present study was to determine the impact of COVID-19-induced home confinement on perceived stress, cognitive aspects, and lifestyle behaviours and the correlations between them.

## 2. Materials and Methods

### 2.1. Participants

A sample of 144 students at a Saudi Arabian university participated in this study. They were aged between 18 and 22 years (mean age 19.3 ± 1.8 years). Most participants were female (n = 90, 62.5%) and single (n = 129, 89.5%). All participants replied to the online questionnaire and completed a cognitive performance test (d2 test) before and during 75 days of confinement. At each assessment, the measures were completed over a one-week period. Psychological and cognitive data were collected through an online survey and via GoogleMeet. The survey and cognitive test were initially sent via emails and then thoroughly explained to students through GoogleMeet by an expert researcher.

The survey comprised of a battery of questionnaires. Initially, participants reported demographic information. Then they completed the “Simple Lifestyle Indicator Questionnaire” (SLIQ) and the “Perceived Stress Scale” (PSS). Finally, they completed the d2 test while being monitored by one of three teachers via GoogleMeet. All participants completed both the questionnaires and the cognitive test. Local institutional ethical approval was provided for this study, which was conducted in accordance with the 1964 Declaration of Helsinki. All participants gave their consent to participate in this study by signing a consent form.

Of note, we have included only participants who adhered to government guidelines and protective measures during the confinement, such as curfew and restrictions; suspension of prayers in all mosques in the city; the banning of travel, sporting events, and social gatherings; the closing of all amusement parks, entertainment zones, and restaurants; etc. More specifically, the socio-demographic questionnaire included an item concerning compliance with government guidelines.

### 2.2. Simple Lifestyle Indicator Questionnaire

Lifestyle was assessed using the SLIQ in its adapted and modified version [17]. The SLIQ is a health measurement scale, which comprises of five dimensions, including dietary habits, physical activity, alcohol ingestion, smoking, and stress. The diet and physical activity dimensions are made up of three questions each. Alcohol, smoking, and life stress components have one question each. Raw scores can be calculated for each lifestyle dimension; for example, the diet raw score is the sum of three questions, concerning consumption of vegetables, fruits, and grains and their uptake frequency, which are each scored from 0 to 5. Then, raw scores can be categorized from zero to two (0 = score 0 to 5, indicating poorly dietary habits, 1 = score 6 to 10, indicating an intermediate healthy diet, and 2 = score 11 to 15, indicating healthy dietary habits). This categorized score is known as the “diet category score”. Questions related to physical activity explore the type, intensity (light, moderate and vigorous), and frequency of physical activities practiced by the individual. Physical activity-related scores can be categorized into 0 (unhealthy lifestyle for physical activity), 1 (intermediate lifestyle for physical activity) and 2 (healthy lifestyle for physical activity). This categorized score is known as the “physical activity category score”. Alcohol ingestion-related questions explored alcohol consumption in terms of alcoholic drinks ingested per week. The raw score can be converted into the “alcohol category score” applying the following formula: in the case of 14 or more drinks per week, the score is coded as zero and indicates unhealthy drinking habits; in the case of 8–13 weekly drinks, the score is categorized as 1 and indicates an intermediately healthy lifestyle for alcohol ingestion; and finally, in the case of 0–7 drinks per week, the category score is 2, indicating healthy lifestyles for alcohol intake. Smoking related questions investigated current and former smoking habits. The score is categorized as 0 if the participant is a current smoker, 1 if the individual is a past smoker, and 2 if the person has never smoked. Finally, life stress is measured on a Likert scale ranging from 1 (“not at all stressful”) to 6 (“very stressful”). Scores from 1 to 2, from 3 to 4, and from 5 to 6 indicate unhealthy, intermediate, and healthy stress lifestyles, respectively.

All these five categorized component scores can be then summed up to provide a final SLIQ score, ranging from 0 to 10 (0 = very unhealthy, 10 = very healthy). However, in the present investigation, to avoid losing some of the variance when the single scores are categorized into only three levels, each domain was analyzed in its raw score. Categorizing, indeed, could not sufficiently capture trends in changes (improvements or worsening) in lifestyle behaviours.

### 2.3. Perceived Stress Scale

Perceived stress was measured using the PSS [18]. Ten items measured the extent to which participants have found their lives unpredictable, uncontrollable, and overwhelming during the last month. The 10 items (six recoded) are rated on a Likert scale ranging from 0 (never) to 4 (very often). The scale shows good convergent and predictive validity with life events, depression, use of healthcare services, and adoption of healthy behaviours [18,19] and has been used frequently in previous research.

### 2.4. Attention Assessment

The d2 test was used to determine the level of concentrated visual attention of participants [20]. It consists of 14 rows with 47 characters per line. These characters are the letters d or p, with a total of one to four dashes above and below each letter. Participants were asked to scan each line and cross out only the characters containing the letter d with two dashes during 20 s. After completion of the d2 test, two variables were calculated: concentration performance (CP) and total number of errors made by the participants (E). CP is calculated as the number of correctly marked d2-symbols minus the number of incorrectly marked symbols (symbols that are not d2-symbols). The total number of E is assessed as the number of errors made by failing to correctly identify a d2-symbol plus the number of errors made by incorrectly marking symbols that are not d2-symbols. We considered both CP and E in the current study.

### 2.5. Statistical Analysis

Descriptive statistical analysis was carried out by computing the means and standard deviations for each of the variables under study. Paired Student’s t-tests assessed differences between before and during confinement. Pearson product-moment correlation tests were used to assess possible relationships between variables during and before confinement. The magnitude of the Pearson’s correlation coefficient was interpreted utilizing the rule of thumb developed by Hinkle et al. [21]; the strength of the correlation was considered negligible if the r coefficient ranged from 0.00 to 0.29, whereas it was deemed low in the range from 0.30 to 0.49, moderate from 0.50 to 0.69, high from 0.70 to 0.89, and very high from 0.90 to 1.00. To shed light on the relationship between variables under study, both mediation and moderation analyses were carried out. All statistical analyses were conducted utilizing the commercial software “Statistical Package for Social Sciences” (SPSS version 24.0, IBM, Armonk, NY, USA), except for the mediation and moderation analyses which were run by means of XLSTAT (version 2020.5.1., Addinsoft, New York, USA). The partial least squares path modeling (PLS-PM) approach to structural equation modeling (SEM) was utilized, in that PLS-SEM is a technique that enables researchers to compute complex models that employ latent variables, being a component-based estimation method. We chose PLS-SEM since, contrary to other techniques, it is particularly flexible, reliable, statistically robust, and can be used also in cases of relatively small sample sizes. Results with *p*-values less than 0.05 were considered statistically significant. All statistical tests were two-tailed.

## 3. Results

During the confinement, only four participants (2.8%) preserved good dietary habits, consuming healthy foods, whereas 100 (69.4%) and 40 (27.8%) ingested poor and unhealthy foods, respectively. In terms of alcohol consumption, all the participants reported consuming a low number of drinks per week. A total of 12 participants did not smoke, 52 were past smokers, and 80 had never smoked. A total of 40 were not stressed at all, 84 were moderately stressed, and 20 highly stressed.

Table 1 reveals that all measured variables significantly varied from before to during confinement (all, *p* < 0.001, with E being significant at *p* = 0.0129). Specifically, CP and activity levels were lower during confinement than before. Errors, total PSS, diet raw score, alcohol raw score, smoking raw score, and stress raw score were higher during confinement compared with before confinement (all, *p* < 0.001).

Before confinement (Table 2), data showed significant correlations between PSS and lifestyle behaviours; more specifically, the correlation was negative and moderate with the diet raw score (r = −0.69, *p* < 0.001), negative and high with the activity raw score (r = −0.84, *p* < 0.001), positive and low with the alcohol raw score (r = 0.39, *p* < 0.001), as well as with the smoking raw score (r = 0.38, *p* < 0.001), and, finally, positive and high with the stress raw score (r = 0.75, *p* < 0.001). In addition, CP was negatively and highly correlated with PSS (r = −0.87, *p* < 0.001) and exhibited different significant associations with lifestyle behaviours, namely, a positive and moderate one with the diet raw score (r = 0.62, *p* < 0.001), a positive and very high one with the activity raw score (r = 0.94, *p* < 0.001), a negative and low one with the alcohol raw score (r = −0.39, *p* < 0.001), as well as with the smoking raw score (r = −0.35, *p* < 0.001), and a negative and high one with the stress raw score (r = −0.85, *p* < 0.001).

Similar but opposite trends could be reported for E, the correlation coefficients of which were low and positive with PSS (r = 0.44, *p* < 0.001) and life stress (r = 0.35, *p* < 0.001) and negative with CP (r = −0.47, *p* < 0.001). The association was deemed moderate and negative with activity (r = −0.51, *p* < 0.001) and negative and low with diet (r = −0.43, *p* < 0.001), and positive but negligible with alcohol consumption (r = 0.20, *p* = 0.016). Finally, the correlation with smoking was computed to be negligible and positive (r = 0.27, *p* = 0.001).

During confinement (Table 3), the data showed significant correlations between PSS and lifestyle behaviours; more specifically, the correlation was negative and negligible with the activity raw score (r = −0.23, *p* = 0.03), whereas it was positive and very high with the diet raw score (r = 0.98, *p* < 0.001), positive and moderate with the alcohol raw score (r = 0.59, *p* < 0.001), and positive and low with the smoking raw score (r = 0.46, *p* = 0.004), as well as with the stress raw score (r = 0.41, *p* < 0.001). In addition, CP was negatively and highly correlated with PSS (r = −0.85, *p* < 0.001) and exhibited different significant associations with lifestyle behaviours, namely, a positive and high one with the diet raw score (r = 0.83, *p* < 0.001), as well as with the activity raw score (r = 0.85, *p* < 0.001), a negative and moderate one with the alcohol raw score (r = −0.67, *p* < 0.001), as well as with the smoking raw score (r = −0.57, *p* < 0.001), and a negative and high one with the stress raw score (r = −0.87, *p* < 0.001).

Similar but opposite trends could be found for E, the correlation coefficients of which were high and positive with PSS (r = 0.76, *p* < 0.001) and life stress (r = 0.71, *p* < 0.001), negative with CP (r = −0.76, *p* < 0.001). The association was deemed moderate and negative with activity (r = −0.53, *p* < 0.001) and diet (r = −0.65, *p* < 0.001), and positive with alcohol consumption (r = 0.53, *p* < 0.001). Finally, the correlation with smoking was computed to be low and positive (r = 0.49, *p* < 0.001).

Focusing specifically on the relationship between PSS and SLIQ (total score), the correlation was high (r = −0.77, *p* < 0.001, Fisher’s z = 1.02) and moderate (r = −0.58, *p* < 0.001, Fisher’s z = 0.66) before and during the confinement, respectively. 

Summarizing before and during the COVID-19 induced confinement, all lifestyle behaviours significantly correlated with PSS, E, and CP, but to various extents, with the specific correlation coefficients shown in Table 2 and Table 3. It was noteworthy that the relationship between PSS and diet score, as well as between life stress and dietary habits, changed both direction and magnitude during versus before confinement.

We also explored whether an increase in perceived stress may lead to a poorer lifestyle and this in turn to lower concentration and whether the worsening lifestyle may act as a moderator or mediator. First, we assessed the dimensionality of the SLIQ (Table 4).

We assessed the uni-dimensionality of our model, as shown in Table 5.

We found that the first hypothesis (a worsening lifestyle to be a moderator) had to be rejected (*p* = 0.517), as shown in Table 6.

A worsening in lifestyle acted as a mediator, with perceived stress resulting in higher alcohol consumption, smoking, and life stress and reduced activity physical activity levels, only partially counteracted/mitigated by improved dietary habits. All this may have led to a reduced CP (Table 7).

## 4. Discussion

To the best of our knowledge, this is one the few available studies to investigate the effect of home confinement on PSS, lifestyle behaviours, and attention, along with their relationships with each other. This study showed that confinement has a negative impact on lifestyle behaviours, such as increased alcohol consumption, increased smoking, and decreased physical activity, and these variables were associated with PSS and attention. However, poorer lifestyles were partially counteracted/mitigated by an increase in healthy food consumption. At the mediation analysis, we found that behavioral lifestyles mediated the association between PSS and CP. These subtle behavioural changes in lifestyle habits may reflect attempts and efforts to adapt to new situational contexts [22].

These findings complement and add to the work of previous authors [23] who studied the effect of COVID-19-induced home confinement in a sample of 1047 participants across different countries (Western-Asia (36%), North-Africa (40%), Europe (21%), and other countries (3%)). The authors showed that home confinement increased physically inactivity (+15.2), social isolation (+71.15%), poor sleep (+12.8%), and unhealthy diet behaviours (+10%). Containment implies a lower possibility of recourse to usually effective adjustment strategies, even though the possibility of recourse to food-centred adjustment strategies and associated stimuli are more frequent (i.e., less physical activity and therefore more fear of gaining weight and more dietary restriction; less social contact and therefore less eating with others, hence the risk of reinforcing rigid and stereotypical eating habits). Finally, anorexia and bulimia are associated with altered cellular immunity [24]; binge eating frequently complicates obesity, which is a major risk factor for a severe form of COVID-19 [2]. This can increase perceived stress and therefore increase the risk of relapse in the adoption of unhealthy lifestyle behaviours.

Such discrepancies may depend on a variety of factors and the methodology adopted (such as study country and design, socio-demographic and cultural characteristics of the sample recruited and recruitment/sampling type).

Confinement in the context of the COVID-19 pandemic represents an important stress factor. It may also be explained that participants were more stressed at the second assessment (during the confinement) because they had been exposed for longer to the pandemic and news of mortalities, etc. A multi-country survey has investigated the effect of COVID-19 induced confinement on psychological states in 1047 participants (54% of which were women) [25]. The study reported that quarantine had a negative impact on mental wellbeing, mood, and feelings, with a statistically significant reduction in the total scores of the mental wellbeing and depressive symptoms questionnaires. Moreover, confinement was associated with an increased risk for developing insomnia symptoms [26,27,28]. Insomnia itself is characterized by repercussions on diurnal functioning, such as fatigue or diurnal drowsiness, attention disorders, depressive symptoms [29], anxiety, and addictive disorders [30]. It should also be noted that a reduction in sleep time was associated with a change in the circadian rhythm, a high level of stress, or both, which in turn can (a) make participants more vulnerable and prone to viral infections [31], (b) increase the risk of psychological disorders [32], (c) have a negative impact on cognitive performance and decision-making, and (d) increase the risk of addiction and impulsivity [33]. Hawryluck et al. [34] observed a 29% increase in moderate symptoms of PTSD in “Severe Acute Respiratory Syndrome” (SARS)-confined individuals upon release from confinement. At the beginning of the COVID-19 pandemic, 4.6% of participants in a sample of 2032 participants from the general population reported a high level of PTSD related symptoms [35].

Self-isolation or confinement may also have a negative effect on physical activity [23]. For instance, Gallo et al. [36] reported that self-isolation reduced activity levels by 30% and increased inactivity (daily sitting time, from 5 to 8 h) compared to the pre-pandemic years [37,38]. Prolonged inactivity may lead to various health issues [36], such as cardiovascular and all-cause mortality [38]. In contrast, another study showed an equal level of physical activity intensity during the lockdown, compared to the pre-pandemic period in citizens of the United Kingdom (20 years old) [39]. Another study [40] reported that 22.4% of active Canadian adults became less active, while 40.3% became more active.

As for the studies on dietary habits, these contradictory findings may be due to the lockdown duration and the transition phase to “normal life” strategies imposed by different governments. The change in direction and magnitude of the correlations between diet and stress may reflect that during confinement participants were using diet manipulation as a coping strategy to help them deal with increased stress because they were being denied access to other coping strategies, such as physical activity or socialization/social support as the majority of participants are single.

The present study showed that confinement has a negative impact on lifestyle behaviours which are also associated with the increase in perceived stress and the decrease in attention. These findings suggest that healthy lifestyle behaviours, including healthy dietary intake, being physically active, not smoking, and not consuming alcohol, were associated with decreased stress and improved cognitive function during the COVID-19 induced confinement. Accordingly, previous studies have also reported that confinement may reduce physical activity and increase unhealthy diet, depression, anxiety, stress [41], negative mood states [42], and negative mental health [43]. Given the importance of physical activity in counteracting the negative impact of a sedentary lifestyle during confinement, various home-based activities are recommended [44].

To date, research suggests that the adoption of proper lifestyle behaviours, particularly diet, disciplined hygiene, and physical activity, boost mental health, psychological states, and immunity to illness. For instance, the World Health Organization (WHO) recommended an appropriate lifestyle, such as maintaining healthy eating habits and practicing home-based exercise to cope with home confinement and to counteract the psychological consequences of COVID-19 [45]. The WHO and other scientific institutions and researchers have recommended that communities should practice different types and intensities of physical activity, such as dancing, body-weight strength and balance training [44,45,46], skipping ropes, walking up and down the stairs [46], amongst others, for 30 min per day for healthy adults and 1 h per day for children with mild to moderate intensity [47] to reduce the negative feelings of confinement.

Despite its importance, the present study is not without any limitations. The main shortcoming is the relatively small sample size recruited. As such, further research in the field is urgently warranted. Another drawback is the fact that we did not assess some variables that could, at least partially, explain our findings. In addition, compliance with government guidelines was self-reported. Based on our results, we concluded that the COVID-19-induced confinement has a negative effect on stress, attention, and lifestyle behaviours. However, this may not be a direct causal connection, and we have to acknowledge that there may be other explanations; e.g., participants may simply be more stressed at the second assessment because they have been exposed for longer to the pandemic and news of mortalities, among others.

## 5. Conclusions

Our study showed that the COVID-19 restrictions had an impact on lifestyles, generally in a negative direction (reduced physical activity, increased smoking and alcohol ingestion, higher stress levels), only partially counteracted by improved dietary habits and significantly impacting cognitive functions. However, based on the above-mentioned limitations and the contrasting findings from the literature, further research is needed.

## Figures and Tables

**Table 1:** Stress, cognitive performance, and lifestyle among participants before and during the confinement.

	Mean ± Standard Deviation	t Statistics	Degrees of Freedom	*p*-Value
E	Before 19.33 ± 15.12 During 22.56 ± 12.08	2.52	143	0.0129
CP	Before 78.58 ± 12.85 During 69.38 ± 13.07	62.43	143	<0.001
PSS	Before 16.61 ± 4.77 During 21.77 ± 5.03	−23.12	143	<0.001
Diet raw score	Before 4.47 ± 2.18 During 6.5 ± 2.72	−10.99	143	<0.001
Activity raw score	Before 13.30 ± 5.26 During 10.22 ± 5.19	8.13	143	<0.001
Alcohol raw score	Before 2.52 ± 1.15 During 3.61 ± 1.04	−13.18	143	<0.001
Smoking raw score	Before 1.05 ± 0.86 During 1.47 ± 0.65	−7.30	143	<0.001
Stress raw score	Before 2.61 ± 1.32 During 3.77 ± 1.34	−17.45	143	<0.001

Abbreviations: CP: concentration performance; E: errors; PSS: Perceived Stress Scale.

**Table 2:** Correlations between stress, cognitive performance, and lifestyle among participants before the confinement.

	E	CP	PSS	Diet	Activity Raw Score	Alcohol	Smoking	Life Stress
E	1							
CP	−0.47 (<0.001)	1						
PSS	0.44 (<0.001)	−0.87 (<0.001)	1					
Diet	−0.43 (<0.001)	0.62 (<0.001)	−0.69 (<0.001)	1				
Activity raw score	−0.51 (<0.001)	0.94 (<0.001)	-0.84 (<0.001)	0.64 (<0.001)	1			
Alcohol	0.20 (0.016)	−0.39 (<0.001)	0.39 (<0.001)	−0.43 (<0.001)	−0.38 (<0.001)	1		
Smoking	0.27 (0.001)	−0.35 (<0.001)	0.38 (<0.001)	−0.44 (<0.001)	−0.36 (<0.001)	0.50 (<0.001)	1	
Life stress	0.35 (<0.001)	−0.85 (<0.001)	0.75 (<0.001)	−0.54 (<0.001)	−0.77 (<0.001)	0.39 (<0.001)	0.32 (<0.001)	1

Abbreviations: CP: concentration performance; E: errors; PSS: Perceived Stress Scale.

**Table 3:** Correlations between stress, cognitive performance, and lifestyle among participants during the confinement.

	E	CP	PSS	Diet	Activity Raw Score	Alcohol	Smoking	Life Stress
E	1							
CP	−0.76 (<0.001)	1						
PSS	0.76 (<0.001)	−0.85 (<0.001)	1					
Diet	−0.65 (<0.001)	0.83 (<0.001)	0.98 (<0.001)	1				
Activity raw score	−0.53 (<0.001)	0.85 (<0.001)	−0.23 (0.03)	−0.10 (0.1)	1			
Alcohol	0.53 (<0.001)	−0.67 (<0.001)	0.59 (<0.001)	−0.51 (0.001)	−0.52 (0.001)	1		
Smoking	0.49 (<0.001)	−0.57 (<0.001)	0.46 (0.004)	−0.55 (<0.001)	−0.46 (0.004)	0.35 (0.03)	1	
Life stress	0.71 (<0.001)	−0.87 (<0.001)	0.41 (<0.001)	0.40 (0.001)	−0.14 (0.03)	0.78 (<0.001)	0.50 (0.002)	1

Abbreviations: CP: concentration performance; E: errors; PSS: Perceived Stress Scale.

**Table 4:** Assessment of the dimensionality (factor structure) of the Simple Lifestyle Indicator Questionnaire (SLIQ).

	F1	F2	F3	F4	F5
Diet	−0.85	−0.14	−0.23	0.44	0.14
Activity raw score	−0.79	0.02	0.61	0.04	0.04
Alcohol	0.79	−0.47	0.17	0.34	−0.13
Smoking	0.69	0.65	0.08	0.31	0.01
Life stress	0.94	−0.19	0.10	−0.08	0.26

**Table 5:** Cross-loadings between stress, concentration performance, and lifestyle among participants during the confinement.

	PSS	SLIQ	CP
CP	−0.85	−0.91	1.00
PSS	1.00	0.80	−0.85
Diet	−0.72	−0.86	0.85
Activity raw score	−0.60	−0.79	0.67
Alcohol	0.59	0.79	−0.68
Smoking	0.47	0.68	−0.57
Life stress	0.82	0.94	−0.89

Abbreviations: CP: concentration performance; PSS: Perceived Stress Scale; SLIQ: Simple Lifestyle Indicator Questionnaire.

**Table 6:** Findings from the moderation analysis between stress, concentration performance, and lifestyle among participants during the confinement.

Latent Variable	Value	Standard Error	t Statistics	Statistical Significance Pr > |t|
SLIQ -> CP	−0.73	0.16	−4.69	0.000
PSS -> CP	−0.37	0.06	−5.69	0.000
Moderation effect	0.12	0.18	0.65	0.517

Abbreviations: CP: concentration performance; PSS: Perceived Stress Scale; SLIQ: Simple Lifestyle Indicator Questionnaire.

**Table 7:** Findings from the mediation analysis between stress, concentration performance, and lifestyle among participants during the confinement.

Latent Variable	Value	Standard Error	t Statistics	Statistical Significance Pr > |t|
PSS -> SLIQ	0.80	0.05	16.02	0.000
PSS -> CP	−0.34	0.05	−6.71	0.000
SLIQ -> CP	−0.64	0.05	−12.59	0.000

Abbreviations: CP: concentration performance; PSS: Perceived Stress Scale; SLIQ: Simple Lifestyle Indicator Questionnaire.

## Data Availability

Data are available upon written request to the Corresponding Author
